# Point prevalence of SARS-CoV-2 infection in Sweden at six time points during 2020

**DOI:** 10.1186/s12879-022-07858-6

**Published:** 2022-11-17

**Authors:** Ramona Groenheit, Jessica Beser, Sharon Kühlmann Berenzon, Ilias Galanis, Edward van Straten, Jan Duracz, Marie Rapp, Disa Hansson, Mikael Mansjö, Sandra Söderholm, Shaman Muradrasoli, Anna Risberg, Richard Ölund, Andreas Wiklund, Kristoffer Metzkes, Matilda Lundberg, Philip Bacchus, Karin Tegmark Wisell, Andreas Bråve

**Affiliations:** 1 Public Health Agency of Sweden, 171 82 Solna, Sweden; 2 European Public Health Microbiology Training Programme, European Centre for Disease Prevention and Control, 169 73 Solna, Sweden; 3 National CBRN Defence Unit, Swedish Armed Forces, 901 76 Umeå, Sweden

**Keywords:** SARS-CoV-2, COVID-19, Point prevalence, Large-scale prevalence surveys, Symptoms

## Abstract

**Background:**

In order to estimate the prevalence and understand the spread of SARS-CoV-2 in Sweden, the Public Health Agency of Sweden, with support from the Swedish Armed Forces, conducted a series of point prevalence surveys between March and December 2020.

**Methods:**

Sampling material and instructions on how to perform self-sampling of the upper respiratory tract were delivered to the homes of the participants. Samples were analysed by real-time PCR, and the participants completed questionnaires regarding symptoms.

**Findings:**

The first survey in the Stockholm region in March 2020 included 707 participants and showed a SARS-CoV-2 prevalence of 2.5%. The following five surveys, performed on a national level, with between 2461 and 2983 participants, showed SARS-CoV-2 prevalences of 0.9% (April), 0.3% (May), 0.0% (August), 0.0% (September), and 0.7% (December). All positive cases who responded to questionnaires reported experiencing symptoms that occurred from 2 weeks before the date of sampling up to and including the date of sampling.

**Interpretation:**

None of the individuals shown to be PCR-positive were asymptomatic at the time of sampling or in the 14 days prior to sampling. This is in contrast to many other surveys in which a substantial proportion of positive cases have been reported to be asymptomatic. Our surveys demonstrate a decreasing ratio between notified cases and the observed prevalence throughout the year, in line with increasing testing capacity and the consecutive inclusion of all symptomatic individuals in the case definition for testing.

**Supplementary Information:**

The online version contains supplementary material available at 10.1186/s12879-022-07858-6.

## Background

The first confirmed case of coronavirus disease 2019 (COVID-19) in Sweden was reported on 31 January 2020 [1]. Since then, multiple independent introductions have resulted in the disease spreading throughout the country, with 2,564,423 reported cases and 19,810 deaths by 31 August 2022 [2].

On 2 February 2020, the Swedish Government included COVID-19 in the Communicable Diseases Ordinance as a disease dangerous to public health and to society, at the request of the Public Health Agency of Sweden [3]. This amendment obliged all individuals and physicians to investigate all suspected cases of COVID-19. The nationally recommended prioritizing indications for testing of COVID-19 were revised several times throughout 2020 according to the increasing testing capacity [4]. Up until 13 March 2020, all individuals with a travel history to any countries with community transmission according to the WHO and any of the symptoms cough, elevated body temperature, or dyspnoea were sampled. On 13 March 2020, community transmission was declared in Sweden. The need for testing for severe acute respiratory syndrome coronavirus-2 (SARS-CoV-2) then exceeded the national capacity, thus prioritizing of testing to the health care and elderly care sector was recommended at a national level. Simultaneously, all individuals having any kinds of symptoms of respiratory disease or other symptoms consistent with COVID-19 were recommended to stay at home and avoid contact with other individuals. Consequently, individuals in the community without medical indication and not belonging to any of the prioritized groups were, in general, not prioritized for testing. As testing capacity was ramping up, additional groups in society could be included in the prioritized groups for testing according to a national testing strategy [4]. On 11 June 2020 the Swedish government and The Swedish Association of Local Authorities and Regions together agreed that the regions would commit to have testing capacity available to provide testing to all individuals with symptoms of COVID-19. This was achieved 11 July 2020, when all regions had established an adequate testing capacity. An overview of the testing strategy is available at ECDC COVID-19 country overviews [5].

Accurate estimates of the true number of infected individuals over time are needed to parameterize mathematical models of SARS-CoV-2 transmission, which are important tools the Public Health Agency of Sweden uses to plan and evaluate COVID-19 control measures and predict future scenarios. However, given the increasing national testing capacity, we assumed a decrease in the ratio of the true number of cases versus detected/notified cases over time. Therefore, point prevalence and seroprevalence estimates are needed to more accurately estimate the population prevalence at different time points.

Self-sampling of the upper respiratory tract at home or at drive-in test centres is an efficient alternative to sampling by trained health care professionals in health care facilities. With the large-scale global demand and the shortage of personal protective equipment during the early phase of the pandemic the concept of self-sampling was developed to allow for large-scale testing among individuals not in need of medical care. Additionally, this sampling methodology reduces the risk of potentially infected individuals leaving their homes and exposing others to the disease. Moreover, self-sampling at home requires less effort for individuals to participate in surveys, which may increase the level of participation. With these considerations in mind, the Public Health Agency of Sweden together with the Swedish Armed Forces performed a pilot study evaluating a self-sampling methodology [6] and further developed concepts for the distribution of material for self-sampling and the collection of samples, thus enabling nationwide surveys.

Our main aim was to estimate the prevalence of COVID-19 in the population of Sweden and the Stockholm region at different time points in order to provide estimates of the true number of cases to use in mathematical models of the spread of SARS-CoV-2 in the population. A secondary aim was to estimate the proportion of asymptomatic cases in the population and to describe the most common symptoms among those testing positive for SARS-CoV-2.

## Methods

### Survey design and participants

Six population-based cross-sectional surveys (surveys 1–6) were conducted in Sweden between March and December 2020.

Survey 1 was carried out in the Stockholm region from 26 March to 3 April. Surveys 2 to 6 were conducted at the national level during the following time periods: 21–24 April, 25–28 May, 24–28 August, 21–25 September, and 30 November–4 December.

Participants in surveys 1 to 5 were recruited from a pre-existing web panel regularly used for health-related questionnaires at the Public Health Agency of Sweden. The web panel was built in 2015 from a simple random sample of 35,000 individuals from the general Swedish population, and in 2016 (n = 15,000) and 2018 (n = 25,000) top-up samples were drawn by age, sex, and education [7]. At the time of the first survey, the web panel included 4,500 individuals.

Survey 6 was designed as an age-stratified random sample from the general population 16 years and older. The calculated sample sizes for the age groups 16–29 years, 30–59 years, and 60 years and older were 2486, 1257, and 1700, respectively, assuming a point prevalence of 2.0%, 1.0%, and 0.4% and a precision of 0.6%, 0.6%, and 0.3%, respectively, at the 5% significance level [8]. From previous experience, we assumed response rates of 30%, 40%, and 40%, respectively, yielding a total of 15,701 individuals to be invited. Statistics Sweden sent invitations to individuals in survey 6 and provided population figures for 2020 [9].

### Ethics approval and consent to participate

The evaluation was performed as part of the Public Health Agency of Sweden’s assignment to monitor communicable diseases and evaluate infection control measures in accordance with §§ 18 of the ordinance (2021:248) from the Swedish Parliament. For this reason, specific ethical clearance for the surveys described in this manuscript was not required. All methods were carried out in accordance with relevant guidelines, and informed consent was obtained from all participants and/or their legal guardians.

### Procedures

Invitations to potential participants were sent 3–5 weeks before the start of each survey and included instructions on how to perform self-sampling at home, information on how the sample would be collected, and when to fill in the questionnaire. Participants were provided with access to a 13-h/day telephone helpline and signed up for the survey by filling out an online form and providing consent; for those under 16 years of age, the legal guardian signed up. Participation was voluntary and could be withdrawn at any time.

In survey 1, material for self-sampling was delivered to the participant’s home by the Swedish Armed Forces, while material for the other surveys was sent by regular post. For all surveys, the Swedish Armed Forces coordinated and collected the samples at the homes of the participants. The samples were collected the same day as the self-sampling or the day after. The participants were asked to store the samples in a refrigerator until collection.

For surveys 1 to 5, self-sampling material consisted of sterile cotton swabs and one test tube containing 1 ml phosphate-buffered saline. Participants were asked to use one cotton swab to perform a throat swab by scraping the posterior pharyngeal wall for 10–20 s and then swirling the cotton swab in the buffer. A second cotton swab was used to sample the distal nasal cavity through both nostrils, followed by swirling the cotton swab in the buffer. In surveys 1 and 2, saliva was collected in a separate collection tube into which the participants had spat 3–4 times, while the participants in the following surveys had swirled a third cotton swab in their saliva before the swab was swirled in the buffer.

For survey 6, participants received one swab and a test tube containing 0.5 ml sample storage reagent consisting of 0.9% normal saline. A swab was used to sample the posterior pharyngeal wall, the distal nasal cavity, and saliva.

In all surveys, participants younger than 16 years had their sampling performed by a caregiver.

The samples were analysed using real-time RT–PCR assays routinely utilized to diagnose COVID-19. Altogether, three different laboratories performed analyses using different targets for real-time RT–PCR for SARS-CoV-2 [10–13] targeting ORF1ab and N-gene with minor modification of primers. The housekeeping genes hBeta-actin (Thermo Fisher Scientific art.no. 4333762F) or hRNAse P (IDT, RNase P, cat no. 10006603) were amplified in parallel with SARS-CoV-2-specific RT-PCR as the internal control for the nucleic acid extraction procedure as well as to confirm that a sample was taken correctly in terms of swabbing against mucosal surfaces in the nose or throat. Samples were considered invalid when hBeta-actin or hRNAse P signals were undetermined or detected as negative, thus indicating lack of human cell material.

Data on the age, sex, and residential region of the participant were gathered when the sample was collected. In survey 6, the education level of the participants was retrieved from administrative registers. On the day of self-sampling, the participants answered an online questionnaire about specific symptoms experienced in the past 24 h and 2 weeks prior to self-sampling. An additional symptom questionnaire was distributed seven days after self-sampling to those who tested positive for SARS-CoV-2.

The number of reported COVID-19 cases and the number of individuals tested for COVID-19 were retrieved from the Public Health Agency of Sweden.

### Statistical analysis

The point prevalence was calculated as the weighted proportion of individuals testing positive for SARS-CoV-2 among those with valid test results. In surveys 1 to 5, weighting included the sample weights from the web panel cohort, non-response in the survey, and population size by age, sex, and region. In survey 6, the weighting was based on the age, sex, region, and education level of the participants in order to consider the sampling error, non-response rate, and population size. Confidence intervals (CIs) were calculated using the Clopper-Pearson exact method. Estimates were reported by sex and age group (0–15 years, 16–29 years, 30–59 years, 60 years and older) for Sweden and for the Stockholm region. We calculated the proportion of each symptom among those who tested positive for SARS-CoV-2; the proportion and its 95% CI were not weighted. Differences in prevalence between sexes were tested with univariate weighted logistic regression. Analyses were carried out in R v.3.6.2 (R Core Team 2019) [14] and survey package v.4.0 (Lumley, 2020) [15].

## Results

### Description of participants

In survey 1 in the Stockholm region, 738 individuals participated, while in the following five national surveys, between 2,471 and 3,038 individuals participated each time (Additional file 1: Table S1). Participation rates were 55–67% for the first five surveys from the web panel and 19% for survey 6 from the general population. In general, individuals between 16 and 29 years old tended to participate to a lesser degree than other age groups, and males participated to a lesser degree than females (Table 1). The distribution of participants by region followed a similar pattern as the general population.

**Table 1:** Distribution of participants by age group, sex and region by survey in Sweden in 2020

		Survey						Swedish population
		26 March–3 April	21–24 April	25–28 May	24–28 August	21–25 September	30 November–4 December	
		(n = 738) (%)	(n = 2,586) (%)	(n = 2969) (%)	(n = 2,527) (%)	(n = 2471) (%)	(n = 3038) (%)	(n = 10,379,295) (%)
Age group	0–15 years	20.8	18.9	17.2	15.1	15.1	–	18.8
	16–29 years	7.2	6.9	8.2	6.9	6.9	37.8	16.9
	30–59 years	42.3	39.5	38.0	38.0	38.0	26.6	38.6
	60 + years	29.7	34.7	36.6	40.0	40.0	35.6	25.6
Sex	Females	51.6	54.5	53.9	53.3	53.4	55.5	49.7
	Males	48.4	45.5	46.1	46.7	46.6	44.5	50.3
Region	Stockholm	100	26.4	25.7	24.7	25.7	26.5	23.0
	Västra Götaland	–	16.1	16.2	16.1	16.0	17.9	16.7
	Skåne	–	13.7	13.2	13.7	13.8	11.7	13.4
	Östergötland	–	4.6	4.4	4.0	4.2	5.5	4.5
	Uppsala	–	4.3	4.2	3.9	4.1	4	3.7
	Jönköping	–	3.3	3.1	3.6	3.4	3	3.5
	Halland	–	3.3	3.7	3.6	3.4	2.8	3.2
	Södermanland	–	2.8	2.9	3.0	3.0	3.1	2.9
	Örebro	–	2.6	2.6	2.5	2.4	3.2	2.9
	Dalarna	–	2.6	2.6	2.9	2..9	2.6	2.8
	Gävleborg	–	2.6	2.5	2.8	2.6	2.3	2.8
	Västmanland	–	2.4	2.6	2.9	2.8	2.1	2.7
	Värmland	–	1.3	1.9	1.9	1.7	2.3	2.7
	Västerbotten	–	3.0	2.8	3.1	2.9	2.6	2.6
	Västernorrland	–	2.1	2.3	2.2	2.1	1.7	2.4
	Kalmar	–	1.9	1.8	1.7	1.7	1.7	2.4
	Norrbotten	–	1.8	2.0	2.0	1.9	2.3	2.4
	Kronoberg	–	2.4	2.3	2.4	2.4	1.7	1.9
	Blekinge	–	1.2	1.2	1.3	1.2	0.9	1.5
	Jämtland	–	1.0	1.1	1.1	1.0	1.0	1.3
	Gotland	–	0.7	0.8	0.8	0.9	0.7	0.6

In total, 14,329 samples were collected from 6608 individuals. Among the individuals participating, 3545 participated in one survey, 605 in two, 739 in three, 1374 in four, 344 in five, and one person participated in all six surveys. We excluded 132 samples that had invalid test results, thus 14,197 were included in the estimations (Table 2). In addition to the lack of hBeta-actin or hRNAse P, invalid test results were also due to participants failing to properly close the test tube, resulting in leakage, or parts from the swab remaining in the test tube.

**Table 2:** Number of samples with valid test results, number of samples positive for SARS-CoV-2, and weighed population prevalence with 95% confidence intervals at the national level and for the Stockholm region in Sweden in 2020

Survey	Dates of survey	Sweden			Stockholm Region		
		Samples with valid test results	Positive samples	Weighted population prevalence (95% CI)	Samples with valid test results	Positive samples	Weighted population prevalence (95% CI)
		(n = 13,490)	(n = 56)		(n = 4192)	(n = 45)	
1	26 March–3 April				707	18	2.5% (1.4–4.1)
2	21–24 April	2571	23	0.9% (0.6–1.5)	679	12	2.3% (1.1–4.2)
3	25–28 May	2957	9	0.3% (0.1–0.5)	761	5	0.7% (0.2–1.6)
4	24–28 August	2518	0	0.0% (0.0–0.2)	623	0	0.0% (0.0–0.6)
5	21–25 September	2461	0	0.0% (0.0–0.2)	632	0	0.0% (0.0–0.6)
6	30 November–4 December	2983	24	0.7% (0.4–1.2)	790	10	1.0% (0.4–2.1)

### Point prevalence

Overall, 74 of 14,197 samples tested positive for SARS-CoV-2 (Table 2). The weekly prevalence at the national level was highest in April, at 0.9% (95% CI 0.6–1.5), and then it decreased in May to 0.3% (95% CI 0.1–0.5). In August and September, no samples positive for SARS-CoV-2 were found, while the weekly prevalence increased to 0.7% (95% CI 0.4–1.2) in December. The same pattern was seen in the Stockholm region, although the weekly prevalence in April was almost three times higher than the prevalence at the national level during the same week (Table 2).

**Fig. 1:**
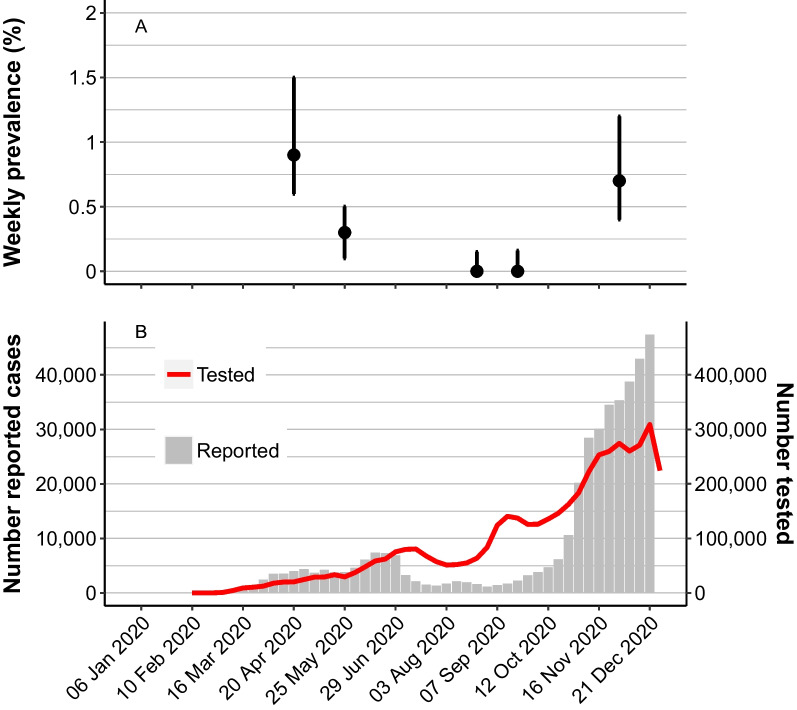
**A** The estimated weekly prevalence of SARS-CoV-2 infection with 95% CIs from the five national population surveys in Sweden in 2020. **B** The number of notified cases of COVID-19 and the number of tested individuals per week in Sweden in 2020

Figure 1 shows the weighted population prevalence estimates as well as the number of newly reported cases and tested individuals per week during 2020. The Public Health Agency of Sweden used a number of sources in order to determine the burden of disease, and this was of particular importance during the initial phase of the outbreak because the testing capacity was not sufficient to meet the demands. If looking only at the number of notified cases, the first wave took place between the middle of March and the end of June. The number of notified cases peaked around the middle of June and coincided with an increase in testing. The second wave, with a much higher reported incidence because many more cases were confirmed compared to the initial wave, started around the middle of September and peaked at the end of the year, and the number of tested individuals increased weekly during that period. While weekly point prevalence estimates in April and December were similar, the reported weekly incidence and the number of tested individuals in December were approximately ten times higher than those in April.

**Fig. 2:**
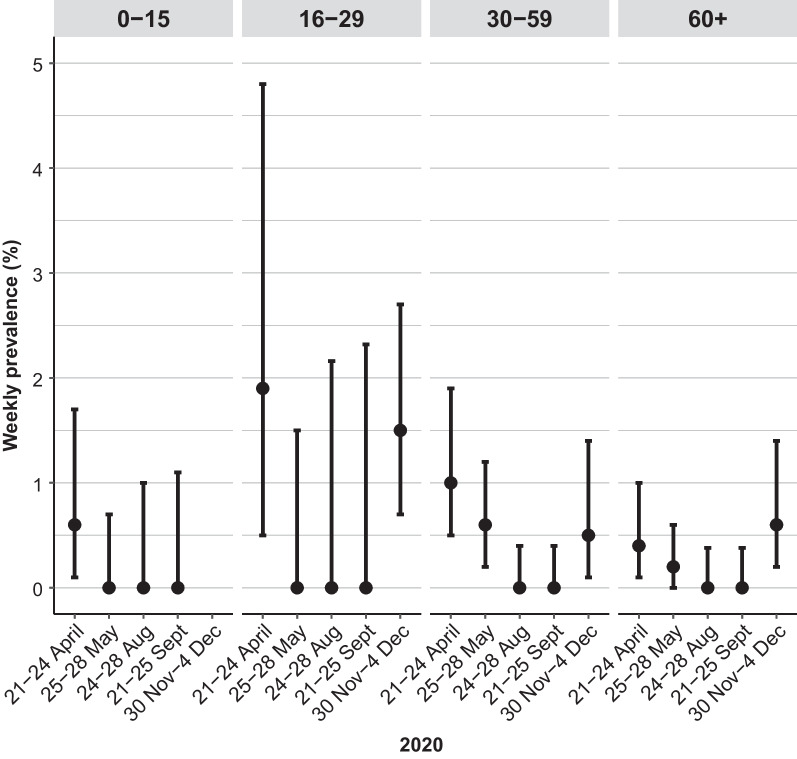
The weighted population weekly prevalence with 95% CIs by age group in Sweden in 2020

At the national level, the highest weekly prevalence among 16–29-year-olds and 30–59-year-olds was seen in April, while among those 60 years and older it was observed in December (Fig. 2, Additional file 2: Table S2). In the Stockholm region, the highest weekly prevalence was observed in the March survey for every age group, with results varying slightly between age groups.

We did not find any significant differences (p > 0.05) between the sexes in terms of the prevalence of SARS-CoV-2 in any of the surveys (Table 3).

**Table 3:** Weighted population prevalence with 95% confidence interval by sex and survey in Sweden in 2020

Survey	Dates of survey	Weighted population prevalence (95% CI)		p value
		Female	Male	
1*	26 March–3 April	3.7% (0.8–7.0)	1.4% (0.1–12.7)	0.07
2	21–24 April	0.7% (0.1–1.7)	1.2% (0.6–2.2)	0.21
3	25–28 May	0.3% (0.1–0.7)	0.2% (0.1–0.6)	0.70
4	24–28 August	0.0% (0.0–0.3)	0.0% (0.0–0.3)	–
5	21–25 September	0.0% (0.0–0.3)	0.0% (0.0–0.3)	–
6	30 November–4 December	1.0% (0.5–1.7)	0.5% (0.2–1.3)	0.26

*Stockholm region

### Positive cases and reported symptoms

Of the 74 individuals positive for SARS-CoV-2, 44 participated in at least one sampling after having tested positive, and none were positive more than once. In total, 72 of the 74 positive individuals answered the first questionnaire about symptoms in the past 24 h and the past 2 weeks (Additional file 3: Table S3). In the 2 weeks prior to self-sampling, 70 of the 72 positive individuals reported having had symptoms, while 69 of the 72 reported having symptoms in the 24 h before self-sampling. All 74 individuals positive for the virus answered the follow-up questionnaire 1 week after self-sampling, and 68 reported experiencing symptoms within those 7 days. All individuals positive for SARS-CoV-2 who responded to the questionnaires reported at least one symptom during at least one of the recall periods.

The most common symptoms in the 2 weeks before self-sampling were runny nose, cough, headache, extreme fatigue, and loss of taste, while 24 h before self-sampling the most common symptoms were headache, runny nose, extreme fatigue, cough and fever, and 1 week later, headache, extreme fatigue, runny nose, cough, and loss of smell (Table 4).

**Table 4:** Proportion of participants positive for SARS-CoV-2 (N = 74) with a 95% confidence interval (95% CI) in six cross-sectional population surveys by reported symptom and recall period in Sweden in 2020

Symptom	Recall period					
	2 weeks before self-sampling (n = 72)		24 h before self-sampling (n = 72)		1 week after self-sampling (n = 74)	
	%	(95% CI)	%	(95% CI)	%	(95% CI)
Chills	13.9	(6.9–24.1)	43.1	(31.4–55.3)	18.1	(10.0–28.9)
Cough	50.0	(38.0–62.0)	62.5	(50.3–73.6)	45.8	(34.2–58.0)
Diarrhoea	18.1	(10.0–28.9)	25.0	(15.5–36.6)	16.7	(8.2–27.0)
Ear pain	6.9	(2.3–15.5)	12.5	(5.9–22.4)	8.3	(3.1–17.3)
Extreme fatigue, exhaustion	41.7	(30.2–53.9)	63.9	(51.7–74..9)	52.8	(40.7–64.7)
Eye discharge	15.3	(7.9–25.7)	16.7	(8.9–27.3)	16.7	(8.9–27.0)
Fever	19.4	(11.1–30.5)	54.2	(42.0–66.0)	20.8	(12.2–32.0)
Headache	48.6	(36.7–60.7)	77.8	(66.4–86.7)	52.8	(40.7–64.7)
Joint pain	20.8	(12.2–32.0)	37.5	(26.4–49.7)	25.0	(15.5–36.6)
Loss of smell	27.8	(17.9–39.6)	37.5	(26.4–49.7)	45.8	(34.2–58.0)
Loss of taste	34.7	(23.9–46.9)	37.5	(26.4–49.7)	43.1	(31.4–55.3)
Myalgia	25.0	(15.5–36.6)	40.3	(28.9–52.5)	30.6	(20.2–42.5)
Nausea	18.1	(10.0–28.9)	31.9	(21.4–44.0)	18.1	(10.0–28.9)
Nosebleeds	5.6	(1.5–13.6)	13.9	(6.9–24.1)	8.3	(3.2–17.3)
Runny nose	58.3	(46.1–69.9)	65.3	(53.1–76.1)	50.0	(38.0–62.0)
Shortness of breath,difficulty breathing	11.1	(4.9–20.7)	20.8	(12.2–32.0)	31.9	(21.4–44.0)
Skin rashes such as hives, dots, pustules or blisters	6.9	(2.3–15.5)	6.9	(2.39–15.5)	5.6	(1.5–13.6)
Sore throat	23.6	(14.4–35.1)	48.6	(36.7–60.7)	31.9	(21.4–44.0)
Stomach ache	18.1	(10.0–28.9)	31.9	(21.4–44.0)	19.4	(11.1–30.5)
Vomiting	2.8	(0.3–9.7)	4.2	(0.9–11.7)	4.2	(0.9–11.7)
No symptoms	4.2	(0.9–11.7)	2.8	(0.3–9.7)	5.6	(1.5–13.6)

## Discussion

We conducted six cross-sectional surveys in the Swedish population to estimate the weekly prevalence of SARS-CoV-2 infection between April and December 2020. The point prevalence estimates varied between 0.0 and 0.9%, with high levels during the first wave in the spring and again in the second wave in the autumn. The Stockholm region generally had higher point estimates than the country as a whole.

While the estimated weekly prevalence was similar in early spring (April) and at the end of the year (December), the reported incidence according to notified cases was several times higher in December compared to April. These observed differences between the estimated weekly prevalence in our surveys and the weekly reported incidence are in line with the increasing testing capacity throughout the year. The proportion of unreported cases can thus be assumed to have decreased during the year, with a higher proportion of unreported cases during the first half of 2020. It is for this reason that it was vital to have prevalence estimates at different time points. Our results were used to calibrate mathematical transmission models for predicting scenarios of the future spread of SARS-CoV-2 in the population at different times during the year [16]. Without the surveys, the models would have had to rely on the number of reported cases alone, which clearly would have affected the accuracy of the output from the models and underestimated the spread of SARS-CoV-2. The proportion of infections that are asymptomatic has not yet been fully elucidated. A meta-analysis of 13 studies with follow-up of symptoms found the proportion of asymptomatic cases to be 17% [17], but various figures ranging from 4 to 100% have been reported [17–25]. Studies aimed at establishing SARS-CoV-2 transmission potential from the proportion of asymptomatic infection generally face two challenges. First, studies relying solely on reported symptoms at one specific time point cannot distinguish between asymptomatic and pre- or post-symptomatic SARS-CoV-2 infection [24]. Second, the requisite for asymptomatic categorization varies greatly, while some studies include all symptoms, others have only one specified symptom [25]. Combined, these challenges illustrate the uncertainty that exists in many studies. In contrast to the surveys conducted in England [26], where a substantial proportion (45–68%) of the infected individuals reported no symptoms around their sampling date, our surveys showed that all positive cases that answered the symptom questionnaire had experienced symptoms within the 2 weeks before sampling. Our timeframe for identifying symptoms, i.e. within 2 weeks prior to sampling and 1 week follow-up for those testing positive, could explain the difference compared to other studies using either no follow-up period or a period prior to the testing [17]. That is, we reduced the possibility that a case could be defined as asymptomatic by considering a broader range of symptoms over a longer duration and by limiting our definition of an asymptomatic case to one who reported none of these symptoms over that timeframe. This discussion highlights the risk of categorizing infected cases as asymptomatic, instead of pre-symptomatic, which in turn can lead to wrongly formulated counter-pandemic strategies. The low number of individuals positive for SARS-CoV-2 in our surveys hampered our ability to conduct several planned comparisons with respect to reported symptoms, including changes over time, differences by age and sex, and negative vs. positive test results.

The surveys were conducted using self-sampling at home, a concept developed and evaluated by the Public Health Agency of Sweden with support from the Swedish Armed Forces [6]. The Public Health Agency of Sweden was granted support from the Swedish Armed Forces in accordance with the regulation regarding Swedish Armed Forces support to civilian authorities. Overall, participants seemed positive about the use of this approach because it allowed them to participate without leaving their home. This was an important aspect because the national recommendations in Sweden included that symptomatic individuals should avoid leaving their homes and, of importance for the results presented here, we did not want to exclude any individuals with symptoms. Sampling at home prevented potentially pre-symptomatic individuals from exposing other individuals to the virus. In contrast to surveys performed in England [26], where study workers monitored self-swabbing in the homes of participants, our participants performed the swabbing on their own following instructions included in the sampling kit. The few samples that had to be excluded due to lack of hBeta-actin or hRNAse P indicate that both adults and caregivers to children were successful in performing the sampling. In the surveys, we used a combination of samples from the throat, nose, and saliva because this showed the highest sensitivity in our pilot study [6].

A strength of our surveys is that we collected information on symptoms experienced within the 2 weeks and 24 h before sampling and, importantly, before the participants knew if they were positive for SARS-CoV-2. It is favourable to ask about symptoms before an individual knows the test result because the knowledge of a positive test can lead to over-interpretation of symptoms [27]. Asking for information about symptoms up to 2 weeks [28] before sampling, not only on the day of sampling, also reduces the risk of misclassifying cases as asymptomatic cases. Issues with recall bias, however, need to be considered when interpreting the results.

For the first five surveys, participants were invited from a pre-existing web panel [7]. This web panel was readily available, and individuals could quickly be invited to join our surveys. Web panel participation rates in the prevalence surveys were just above 50%, which is lower compared to studies conducted with the same panel during 2020 (80–90%) [29]. A weakness of survey 6 was the low participation rate of 19%. A requirement of the prevalence surveys was for participants to self-sample using swabs, as well as to be available at their home address during a specific day and time, albeit chosen by them, for sample collection. This additional effort may have discouraged individuals from participating. Although we applied survey weights to account for non-response, bias due to other unknown factors may remain. Additionally, those who previously had been positive for SARS-CoV-2 may have been less willing to participate even though the invitation encouraged individuals to participate regardless of previous infection or the presence of antibodies.

## Conclusion

We estimated the prevalence of SARS-CoV-2 during 2020 in six cross-sectional national surveys in Sweden with the aim to estimate the national prevalence and the proportion of asymptomatic cases. The results showed that the estimated weekly prevalence was similar in early spring (April) and at the end of the year (December). However, the reported incidence of COVID-19 according to the number of notified cases was approximately ten times higher at the end of the year, meaning that the fraction of unreported cases decreased during the second wave of infections compared to the first wave. Furthermore, by asking participants about the presence of a wide range of symptoms over a 2-week period before and 1-week period after testing, we found that none of the survey participants who tested positive for SARS-CoV-2 were asymptomatic, an important finding that can generally be attributed to how and when information on symptoms was collected.

Our results highlight the need for community prevalence estimates that are independent of the general testing capacity and policies. As seen in Sweden, and in most countries around the world, the testing capacity for SARS-CoV-2 varied over time in line with the available laboratory capacity and the allocated resources during the pandemic. Conducting national surveys was key to obtaining a better estimate of the spread of SARS-CoV-2 in the population.

## Supplementary Information

**Additional file 1: Table S1.** Number of invited individuals, number of participants and participation rate by survey at the national level and for the Stockholm region in Sweden in 2020.

**Additional file 2: Table S2.** Weighted population prevalence with 95% confidence interval by age group in Sweden in 2020.

**Additional file 3: Table S3.** Number of participants positive for SARS-CoV-2 reporting symptoms by recall period in Sweden in 2020.

## Data Availability

The datasets generated and analysed during the surveys are not publicly available due to existing general data protection rules and official secrecy, but they are available from the corresponding author on reasonable request.
