# Supplementary material for: Point prevalence of SARS-CoV-2 infection in Sweden at six time points during 2020
Source: BMC Infect Dis. 2022 Nov 17;22:861. doi: 10.1186/s12879-022-07858-6 (PMC9672540; doi:10.1186/s12879-022-07858-6)
Supplement: Supplementary file 1 — Additional file 1: Table S1. Number of invited individuals, number of participants and participation rate by survey at the national level and for the Stockholm region in Sweden in 2020. [file 12879_2022_7858_MOESM1_ESM.docx]

**Supplementary Table 1.** Number of invited individuals, number of participants and participation rate by survey at the national level and for the Stockholm region in Sweden in 2020.

|  |  | **Sweden** | | |  | **Stockholm Region** | | |
| --- | --- | --- | --- | --- | --- | --- | --- | --- |
| **Survey** | **Dates of survey** | **Invited individuals** | **Participants** | **Participation rate** |  | **Invited individuals** | **Participants** | **Participation rate** |
| 1^*^ | 26 March–3 April |  |  |  |  | 1,106 | 738 | 66.7% |
| 2 | 21–24 April | 4,480 | 2,586 | 57.7% |  | 1,115 | 685 | 61.1% |
| 3 | 25–28 May | 4,487 | 2,969 | 66.2% |  | 1,109 | 772 | 69.6% |
| 4 | 24–28 August | 4,491 | 2,527 | 56.3% |  | 1,115 | 626 | 56.1% |
| 5 | 21–25 September | 4,492 | 2,471 | 55.0% |  | 1,110 | 633 | 57.0% |
| 6 | 30 November–4 December | 15,701 | 3,038 | 19.3% |  | 3,546 | 803 | 22.6% |

^*Stockholm region^
