# Supplementary material for: Point prevalence of SARS-CoV-2 infection in Sweden at six time points during 2020
Source: BMC Infect Dis. 2022 Nov 17;22:861. doi: 10.1186/s12879-022-07858-6 (PMC9672540; doi:10.1186/s12879-022-07858-6)
Supplement: Supplementary file 2 — Additional file 2: Table S2. Weighted population prevalence with 95% confidence interval by age group in Sweden in 2020. [file 12879_2022_7858_MOESM2_ESM.docx]

**Supplementary Table 2.** Weighted population prevalence with 95% confidence interval by age group in Sweden in 2020.

| Survey | Dates of survey | Weighted population prevalence (95% CI) | | | |
| --- | --- | --- | --- | --- | --- |
|  |  | **0**–**15 years** | **16**–**29 years** | **30**–**59 years** | **60+ years** |
| 1^*^ | 26 March–3 April | 2.8% (0.8–7.0) | 2.4% (0.1–12.7) | 2.6% (1.1–5.1) | 2.0% (0.5–5.0) |
| 2 | 21–24 April | 0.6% (0.1–1.7) | 1.9% (0.5–4.8) | 1.0% (0.5–1.9) | 0.4% (0.1–1.0) |
| 3 | 25–28 May | 0.0% (0.0–0.7­) | 0.0% (0.0–1.5) | 0.6% (0.2–1.2) | 0.2% (0.0–0.6) |
| 4 | 24–28 August | 0.0% (0.00–1.00) | 0.0% (0.00–2.16) | 0.0% (0.00–0.40) | 0.0% (0.00–0.38) |
| 5 | 21–25 September | 0.0% (0.00–1.10) | 0.0% (0.00–2.32) | 0.0% (0.00–0.40) | 0.0% (0.00–0.38) |
| 6 | 30 November–4 December |  | 1.5% (0.7–2.7) | 0.5% (0.1–1.4) | 0.6% (0.2–1.4) |

^*Stockholm region^
