# Supplementary material for: Point prevalence of SARS-CoV-2 infection in Sweden at six time points during 2020
Source: BMC Infect Dis. 2022 Nov 17;22:861. doi: 10.1186/s12879-022-07858-6 (PMC9672540; doi:10.1186/s12879-022-07858-6)
Supplement: Supplementary file 3 — Additional file 3: Table S3. Number of participants positive for SARS-CoV-2 reporting symptoms by recall period in Sweden in 2020. [file 12879_2022_7858_MOESM3_ESM.docx]

**Supplementary Table 3.** Number of participants positive for SARS-CoV-2 reporting symptoms by recall period in Sweden in 2020.

| Survey | Dates of survey | Recall period | | |  | |
| --- | --- | --- | --- | --- | --- | --- |
|  |  | **2 weeks before self-sampling** | **24 hours before self-sampling** | **1 week after self-sampling** | | **Total number of participants positive for SARS-CoV-2** |
| 1^*^ | 26 March–3 April | 17 | 18 | 17 | | 18 |
| 2 | 21–24 April | 23 | 20 | 18 | | 23 |
| 3 | 25–28 May | 9 | 9 | 9 | | 9 |
| 4 | 24–28 August | 0 | 0 | 0 | | 0 |
| 5 | 21–25 September | 0 | 0 | 0 | | 0 |
| 6 | 30 November–4 December | 21 | 22 | 24 | | 24 |

^*Stockholm region^
